# New alginate-gelatine method for casting of staining inside firearm barrels

**DOI:** 10.1007/s00414-024-03213-3

**Published:** 2024-03-23

**Authors:** Christian Schyma, Matthias Berthold

**Affiliations:** Institute of Forensic Medicine, University of Bern, Murtenstrasse 26, Bern, CH-3008 Switzerland

**Keywords:** Firearms, Ballistics, Shot range, Biological traces, Molecular genetics, Forensic science

## Abstract

Contact shots to the head often leave behind biological traces inside firearm barrels, a phenomenon of great forensic interest. Until now, the visualization and preservation of these traces presented a significant challenge, lacking a reliable method. This study addresses this gap by searching for a suitable method to extract the traces within a casting. Using alginate or gelatine as suitable materials, the results were hampered by serious adhesion issues and their extraction out of the firearm barrel was impeded. Finally, the combination of 11% gelatine with 1% alginate, introduced into the barrel around a ‘central spine’, succeeded to consistently produce replicable castings. Experimental contact shots displayed a distinct staining gradient from the muzzle to the rear of the barrel, as revealed through endoscopy and proved in the macroscopic casting. The technique proved effective for various common handgun barrels and successfully preserved blood and gunshot residue (GSR) patterns within the barrel. This method offers the dual benefits of visually mapping staining patterns and securing localized samples for targeted molecular genetic analysis in forensic investigations.

## Introduction

After contact shots to the head, traces of blood and tissue debris were detected inside firearm barrels [1–3]. The presence of such traces was documented by endoscopy of the firearm barrel [4, 5]. Using PCR amplification, it was possible to identify the victim from swabs gathered on the inner surface of the barrel [6, 7]. To investigate the mechanism of staining, experimental studies were performed using the triple contrast method [8] in various target models. The so-called reference cube, a gelatine cube with 12 cm edge length, was inseparably covered by a kitchen wipe beneath which a thin foil bag containing a mixture of blood and acrylic paint was embedded [9]. Contact shots generated visible staining along the inner surface of the barrel. Endoscopy of the barrel revealed that the amount of traces decreased from the muzzle to the rear end of the barrel [10]. Swabs gathered separately from the anterior and the posterior part of the barrel were submitted to PCR amplification. The yield of DNA was always higher in the anterior part of the barrel next to the muzzle matching the visual findings [10]. This gradual distribution decreasing from the muzzle to the rear end was reported as well in an early study using only blood [11] as well in studies based on the reference cube [9, 12].

Endoscopy of firearm barrels in real cases of contact shots showed principally the same distribution pattern as in experimental studies [7], however the detection of biological traces is limited by optical resolution and contrast. The idea of “mapping” the staining inside firearm barrels required another approach. The entire pattern of traces had to be extracted from the barrel to allow for visualisation at microscopic scale. The aim of this study was to find a technique of casting the firearm barrel.

In order to extract a cylinder containing most of the trace material, we assessed different materials and their transition into solid state. Ideally, this method should be able to extract both inorganic residues such as gunshot residues (GSR) as well as organic residues like powder particles and DNA-containing material like blood or tissue of the injured body. Furthermore, the casting cylinder must be water-soluble after the extraction, in order to allow for molecular-biologic analysis identifying the victim by DNA-profiling and the tissue by microRNA.

## Materials

First, we screened a panel of materials to identify materials with suitable characteristics. The search lead us to very common and easily accessible materials such as alginate and gelatine, but also other, more expensive materials mostly used in the dental medicine to perform elastic impressions of the denture, were explored, such as polysilicones, polyether and mixtures like vinylsiloxanether. Beside technical and economic aspects, the characteristics of the material were important, such as water solubility, fracture strength and an easy to perform transformation of fluid to solid state. Water solubility is the most important trait, as we aimed to tie biological traces such as blood and tissue to our moulding matrix as well as to dissolve it afterwards. A good fracture strength was needed to get the cylinder undamaged out of the firearm barrel.

Furthermore, a useful object as central spine was searched to extract the moulding matrix from the barrel. For this purpose, different materials (pipe cleaner, twist drills, wooden cotton buds, metal chains and knitting needles) were tested. Finally, a long cotton swab with wooden rod had overall the best qualities in combination with subsequently tested matrices.

### Development of the method

#### Ordnance gelatine

Ordnance gelatine is well known as a simulant in wound ballistics studies and commonly prepared in 10% or 20% solutions.

The gelatine powder (*Ballistic III*, Gelita, Eberbach, Germany) was dissolved in heated purified water. The powder was easily dissolved in temperatures between 40–50° Celsius. No solid state was reached within a useful time range without addition of admixtures regardless of concentration and temperature change. Admixtures were added in order to accelerate or yield a transition to solid state. As admixtures formaldehyde and propanol were tested with the latter being clearly superior in the ability to lead to this transformation. This method led to promising cylinders with good fracture strength, no shrinking effect and good adhesive characteristics. As we proceeded to the trials in firearm barrels, the adhesion was proving to be an insurmountable obstacle, as it was impossible to extract the cylinder once it was solidified. Different methods of extraction as well as different concentrations of propanol and gelatine were tested to improve mobility in the solidified state. Higher concentrations of propanol the surface of the solid gelatine led to an improved mobilization, however the moulding also became smeary and the cylinder lost its texture during the extraction.

#### Sodium alginate

Alginates are salts of alginic acid, a natural polysaccharide found in cell walls of brown algae. When hydrated, the alginate powder transforms into a viscous, hydrophilic gel matrix with high flexibility.

Due to its hydrophilic assets, sodium alginate is great in terms of water solubility. However, its fracture strength is rather weak.

Sodium alginate powder (Fluka, Buchs, Switzerland) was dissolved in purified water at room temperature. Water was purified in-house with a Milli-Q water system from Millipore (Zug, Switzerland). Sodium alginate solution cannot be transformed into solid state by easily practicable methods like e.g. drying. To perform a transition from fluid to solid we made use of a chemical reaction between solute sodium alginate and solute calcium chloride (CaCl_2_). The solute sodium alginate exchanges the Na^+^ ion with the Ca^2+^ ion from the solute calcium chloride in a chemical reaction, in which additional sodium chloride (NaCl) results. The emerged calcium alginate shrinks and releases water, which leads to solid state. Trials with other calcium containing salts – such as calcium silicate – failed.

We tried several methods to add the solute calcium chloride to the solute sodium alginate. Neither adding it by pouring the solutions consecutively nor adding it one or the other outside the tube produced any useful results as the reaction takes place immediately and no cylinder was formed.

Adding the calcium chloride with a syringe in order to add it continuously along the cotton swab was established as the key method and led to reproducible results. The exact concentrations and mass of the mixture between solute alginate and calcium chloride to reach the maximum of fracture strength were determined empirically.

Even with the maximized fracture strength, this trait remained the major weakness, which was exposed when the method was performed in a cleaned firearm barrel. Using a twist drill bit the extraction of a calcium alginate cylinder succeeded, even if blood had been placed in the barrel. However, in trials with barrels of previously fired firearms, the method failed as it was no longer possible to extract the cylinder.

### Alginate-gelatine composition

Both previously described methods had promising traits, but mobilization of the end product failed. Even though the interaction between soluble sodium alginate and soluble gelatine had yet to be explored, rather surprisingly the combination of these substances showed much potential in a pilot experiment, which needed further assessment of the reproducibility and limitations.

The first trials were performed in test tubes. Sodium alginate and gelatine were consecutively dissolved in purified water.

At first, similar to the pilot experiment, solute alginate and solute gelatine were used without any admixtures. As the transition to solid state took a long time, we started to freeze the combination, which led to an accelerated transition.

Next, the admission of the admixtures like propanol or solute calcium chloride – performed similar to the previously described approaches – were tested and neither combination nor the admission of both admixtures simultaneously lead to an useful and reproducible result, despite trying various concentrations and temperature changes for the transition.

As the sole combination of sodium alginate and gelatine showed the highest potential, we kept adapting and improving by varying concentrations from 5% to 20% solute gelatine and 0.5–4% solute sodium alginate. We also varied the time to add the two ingredients together, the time the material stayed in the freezer and the time of unfreezing, which we also tried to accelerate by using a blow dryer. The mobilization out of the test tube was—yet again due to the adhesive characteristic of the gelatine—rather difficult. Various objects as central spine of the casting were tried to further improve the stability of the cylinder during the mobilization. The long wooden cotton buds yet again emerged as the most promising, but yet failing object.

Adding a barrel cleaning plug to create a base area at the cotton bud end enabled reliable pulling and extraction of the casting cylinder and proved to be successful even in firearm barrels.

### Alginate-gelatine method

I) First of all, the firearm barrel is sealed at the rear end using the wooden cotton bud with terminal sub-calibre barrel cleaning plug. Centering of the wooden cotton bud is key, therefor plastilina is used to fix the cotton swab and to complete seal at the rear end. For six inch barrels the cotton swab was too short and substituted by a “trimmed” pipe-cleaner.
II) 15.5 ml purified water is heated to 40–45° Celsius before 200 mg sodium alginate powder is added slowly using a magnetic stirrer. After the sodium alginate is fully dissolved, 2 g gelatine powder is added in a slow rotation. The magnetic stirrer needs to be at an even slower rotation rate after the gelatine is dissolved in order to enable the produced air bubbles to rise to the surface. To further reduce the air bubbles in the liquid, removal of the upper half of the mixture is recommended.
III) The remaining clear solution is carefully poured into the prepared firearm barrel. Promptly afterwards, the filled firearm barrel is put upright into a freezer, at a temperature of minus 25-30° Celsius. The time in the freezer is dependent on the calibre size. For cal. .32 auto (7.65 mm), the freezer time is nine minutes and for 9 mm calibres the freezer time is twelve minutes. For bigger calibre (up to .45) the time is about fifteen minutes and for .22 small calibre the time is about six minutes.
IV) After freezing time, the cylinder is in solid state and needs to be unfrozen before extraction. Again, the unfreezing time is dependent on the calibre. For cal. .32 auto, it is seven minutes, for 9 mm calibres eight minutes. For bigger calibre (.45), the unfreezing time is about nine minutes, and for .22 small calibre the unfreezing time is about six minutes.
V) Afterwards, the extraction is performed with constant slow and sometimes rather strong pull with a slight rotation movement. After the extraction, the cylinder is put on a sterile surface as not to contaminate the end product.

## Results

### Casting of cleaned handgun barrels

**Fig. 1:**
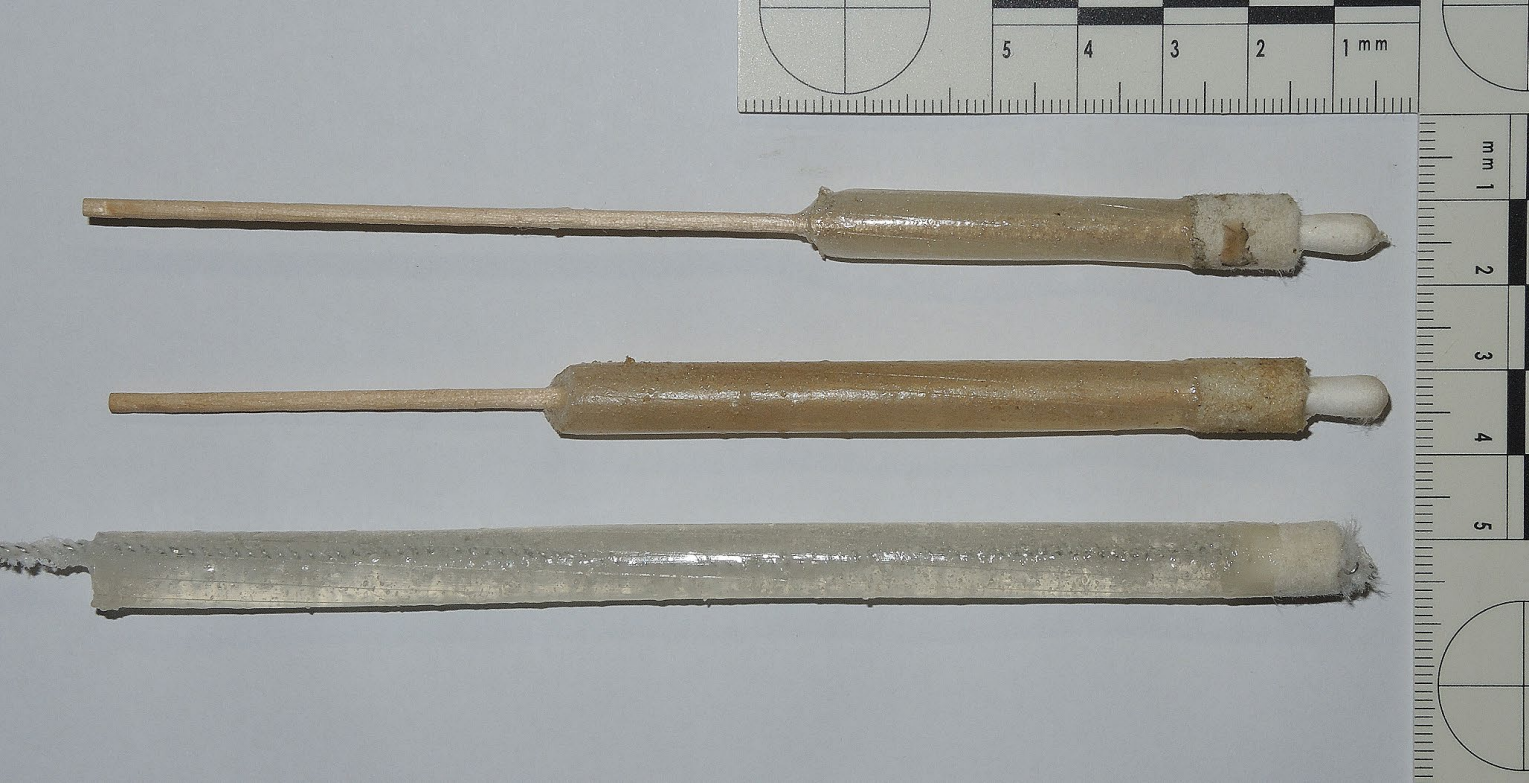
Alginate-gelatine castings from cleaned revolver barrels, muzzle side on the left. From top:.38 special, 1.88 inch barrel (47.7 mm); .38 special, 4 inch barrel (100 mm); .357 Magnum, 6 inch barrel (150 mm)

**Fig. 2:**
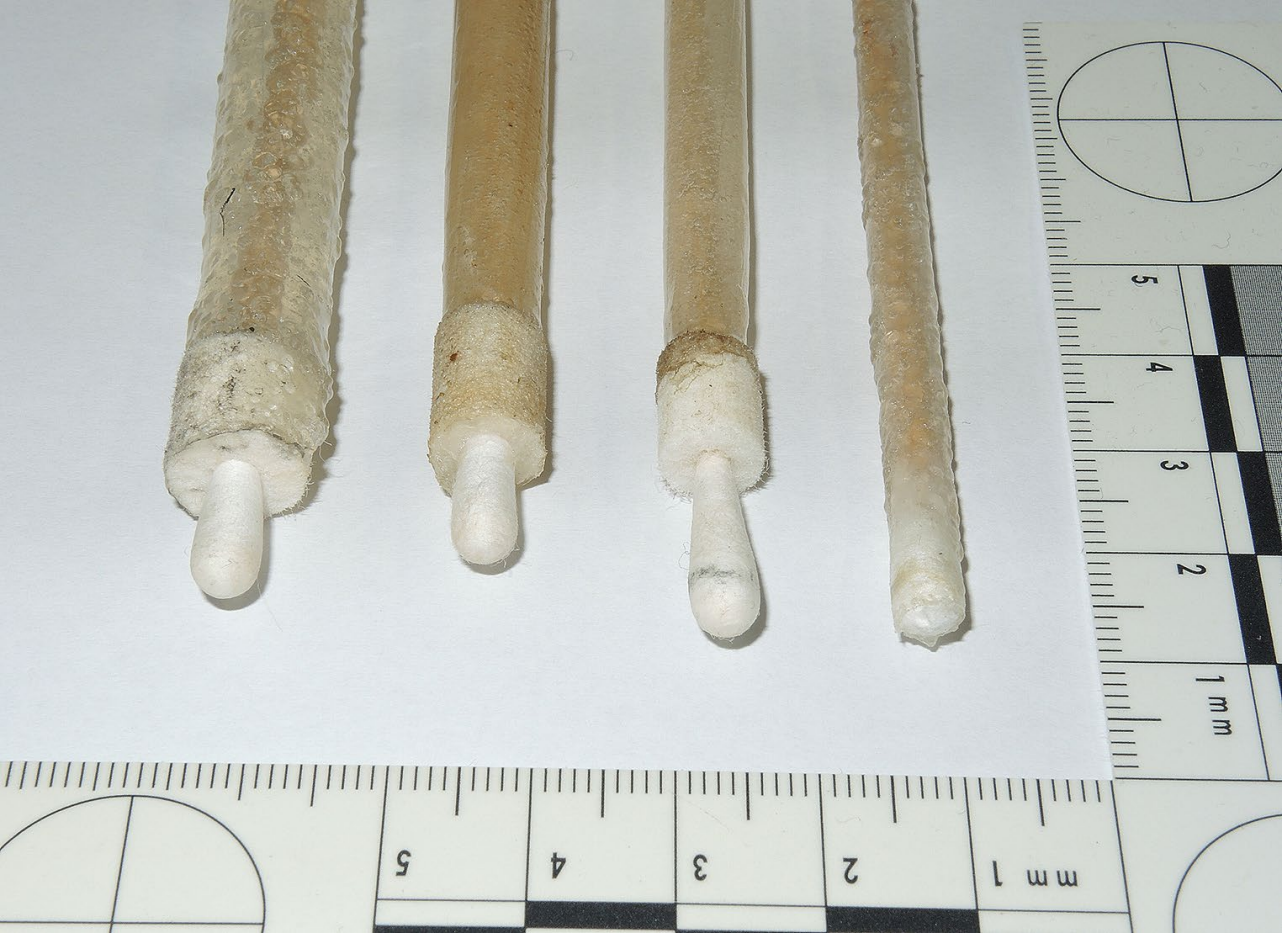
Alginate-gelatine castings from cleaned handgun barrels, the rear of the barrel in the foreground. From left to right: .45 ACP pistol, 9 mm Luger pistol, .32 auto pistol, .22 long rifle revolver

**Fig. 3:**
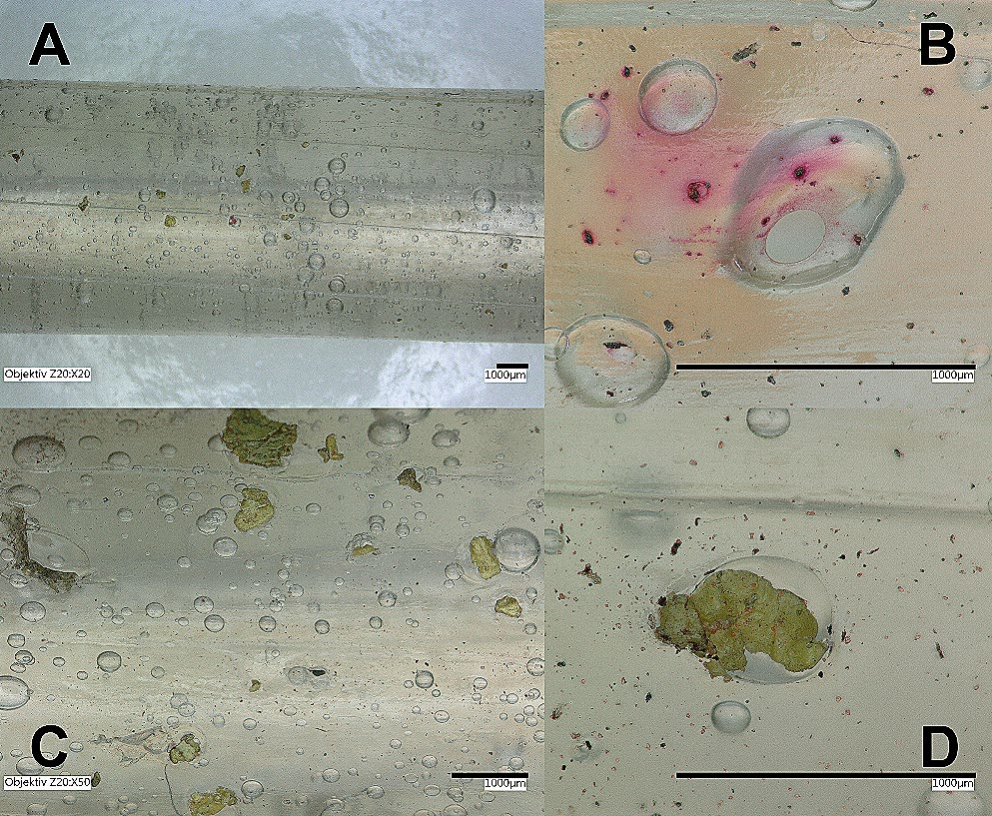
Photographs taken with the digital microscope VHX-5000 (Keyence, Mechelen, Belgium) (**A**) .38 special barrel (20x). (**B**) Rhodizonate positive lead particles (200x). (**C**) Partially burnt powder particles (50x) (**D**) Partially burnt powder particle peppered with GSR (200x)

A selection of current handguns in various calibres was submitted to the alginate-gelatine method: four inch barrelled revolver cal. .22 WMR (Winchester Magnum Rimfire), revolvers cal. .38 special / .357 Magnum with barrel length up to six inch, various types of semi-automatic pistols in .32 auto and 9 mm Luger calibres, .45 ACP pistol mod. 1911. Intact cylindrical castings were extracted up to six inch barrel length (Fig. 1) and from .22 to .45 barrel diameter (Fig. 2). Microscopic inspection showed lands and grooves (Fig. 3A) of the barrel as well as sodium rhodizonate positive GSR (Fig. 3B) or partially burnt powder particles (Fig. 3CD).

### Casting of stained handgun barrels

Contact shots were fired on “reference cubes” [9] doped with heparinised blood of an adult, informed volunteer (according to the approval of the ethics committee of the University Hospital Bonn). Twelve individual firearms (cal. .32 auto, .38 special, 9 mm Luger) were thoroughly cleaned and endoscopically controlled before the shot (non-expanding ammunition). Just after shooting, the staining load was recorded using the endoscopic optic without introducing the endoscope into the barrel. The firearms remained in horizontal position over night.

**Fig. 4:**
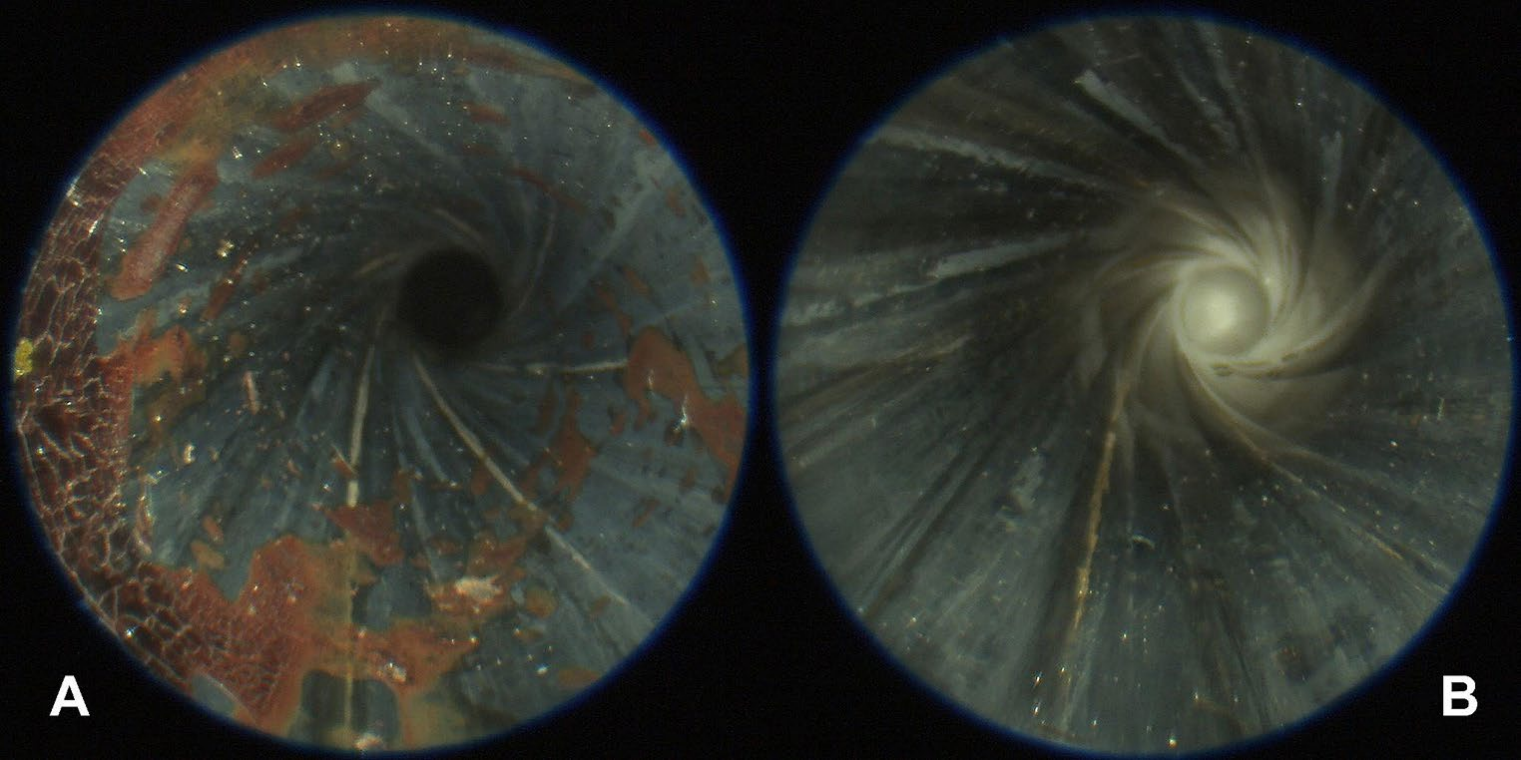
A) Endoscopic insight into the barrel of the Glock pistol cal. 9 mm Luger after contact shot at a reference cube doped with human blood B) Same barrel after removal of the alginate-gelatine casting

**Fig. 5:**
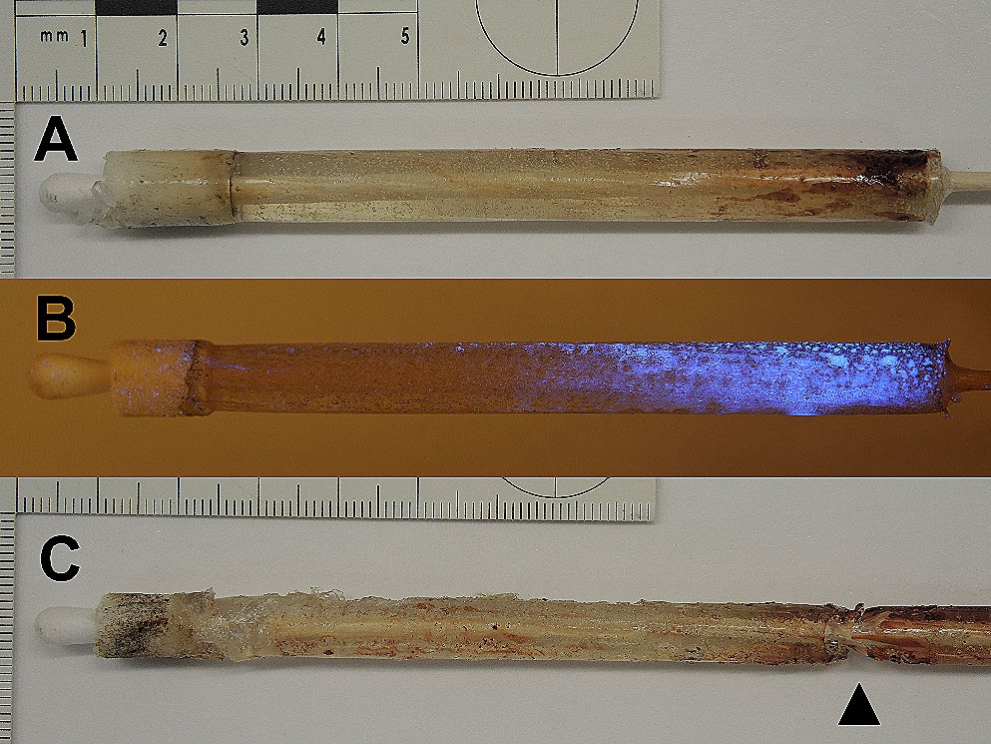
Alginate-gelatine castings of barrels after contact shot at a reference cube. Rear end of the barrel at left, muzzle at the right: A) Pistol Glock 19, cal. 9 mm Luger, barrel length 102 mm B) Revolver Astra Cadix, cal. .38 special, four inch barrel, treated with Luminol C) Pistol FN Mod. 1910/22, cal. 7.65 mm Browning, barrel length 113 mm. Traction artefact (arrowhead)

The next day, after video-endoscopic confirmation of the in situ consolidated traces (Fig. 4A), the alginate-gelatine method was performed (Fig. 4B). It was possible to extract a casting of the barrel containing macroscopic blood traces (Fig. 5A and C) from all twelve handguns. The alginate-gelatine cylinders presented a gradual distribution of the blood traces, which decreased from the muzzle to the rear end (Fig. 5). Barrels with a high load of blood traces tended to castings with rougher surface and traction artefacts, e.g. Walther PP or FN Mod. 1910/22 (Fig. 5C).

Contamination was systematically avoided using surgical masks and gloves. The casting cylinders were stored in Minigrip® bags at -20°C. PCR was successfully performed on targeted samples taken from the alginate-gelatine cylinder. In all analyses, a single STR-profile was obtained that fully matched the donor’s profile.

**Fig. 6:**
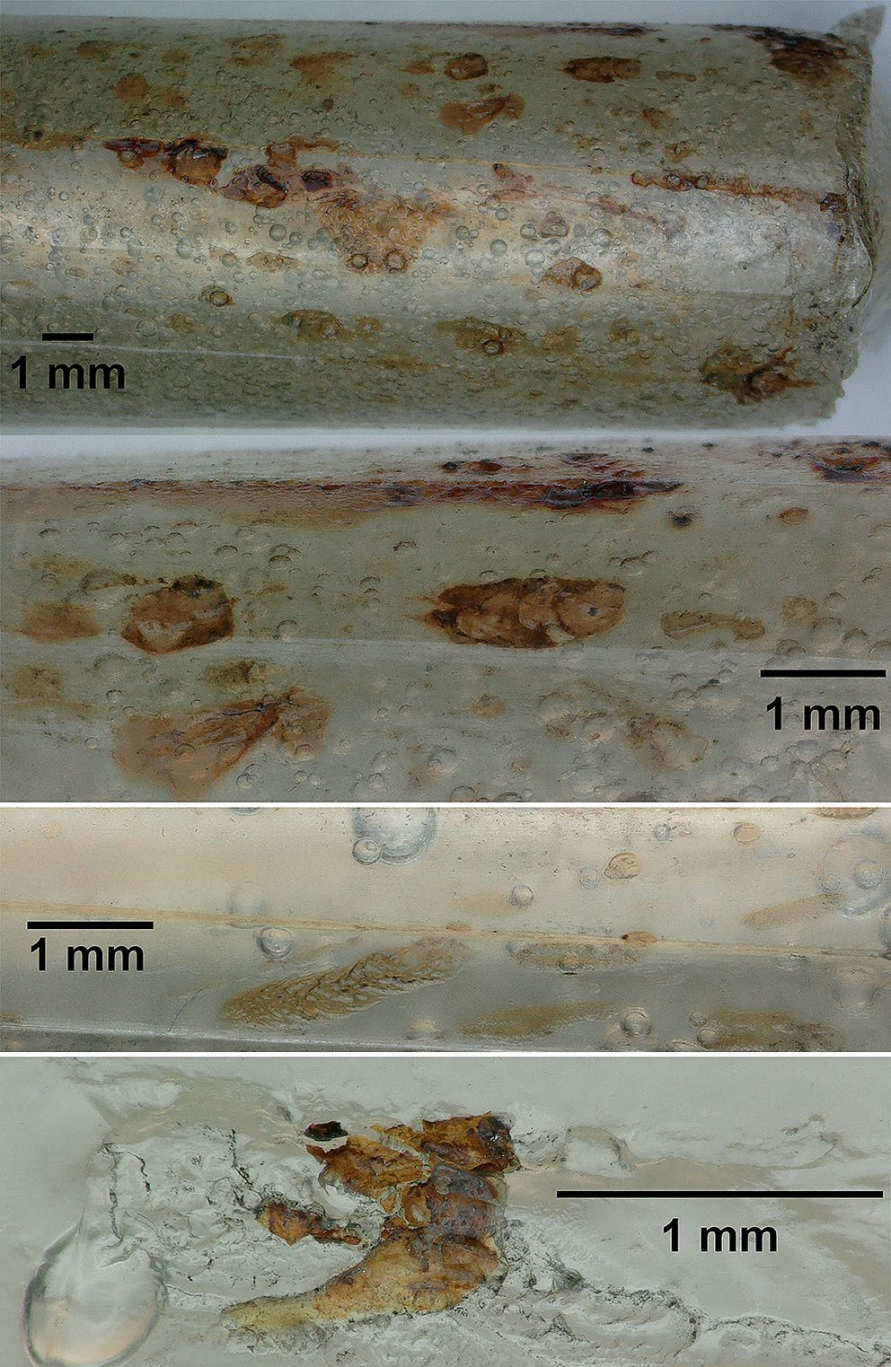
From top: .38 revolver muzzle area. Magnification of embedded blood traces. Fine elongated traces in a .32 auto pistol. Blood with GSR in a Beretta cal. .32 auto

Microscopic examination of the barrel castings demonstrated that the residual small air bubbles in the matrix did not reduce the yield of traces neither affect the integrity of the cylinder surface (Fig. 6). Blood seemed embedded in the matrix without major signs of diffusion. Blood traces were found in various patterns, from tiny spots to spike like forms as well as staining following the grooves (Fig. 6).

## Discussion

The possible presence of biological traces in gunshot barrels, especially after contact shots to the head, inspired research since almost a century [1]. On the one hand, a visual approach using endoscopy assessed morphology and distribution of staining [4–7, 13]. On the other hand, progress in molecular genetics led to identification of the victim [7] as well as tissue origin [14] from swabs of the barrel’s inner surface. Ideally, in forensics, both approaches were combined. Previous research had highlighted the forensic importance of the distribution of traces inside gun barrels for the differentiation of close range shots [15]. Yet, sampling while introducing swabs from both ends of the barrel provided only a rough differentiation between staining in the anterior and posterior part of the barrel.

The idea to “map” the staining inside firearm barrels [15] by endoscopy failed by the inability to provide a reproducible 2-D image of the cylindrical barrel surface because of optical limitations and geometrical conditions. As an alternative, the removal of the entire staining in its topographic distribution was considered. However, the requirements of such an approach were demanding: harmlessness for firearm barrels regarding firearm identification, binding of humid and consolidated traces of blood and tissue into a matrix, compatibility with PCR analysis and conserving GSR for criminal technical analysis. Gelatine and its compatibility to molecular genetic analysis are well known [e.g. 9]. Another candidate for a hydrophilic matrix was alginate that was investigated sixty years ago in the context of electrophoresis and filter membranes [16–18]. Hydrated alginate powder transforms into a viscous gel matrix with flexible properties depending on the diffusion of di- or trivalent cations into the colloid [19].

As both casting methods were tested, it soon became clear that those materials had mechanical properties, which made it difficult to perform a reliable extraction of the casting cylinder. Pure gelatine cylinders adhered too strongly to the barrels inner surface, while alginate tended to react with GSR, which also caused unbreachable adhesion. Even though both methods failed due to excessive adhesion, we assumed that, owing to various factors contributing to this adhesion, a combination of the two materials might potentially work. Indeed, when a solution of 11% gelatine and 1% alginate was introduced into the firearm barrel around a central spine, an intact cylinder containing blood traces as well as GSR could be reliably extracted. This method proved to be successful for common handgun barrels of various calibres and lengths. Traces were preserved in their original topographic location without any signs of displacement during the casting procedure. In the endoscopic control, it was demonstrated that virtually all traces could be extracted from the firearm barrel. Following experimental contact shots, while the quantity of blood traces varied, the staining revealed a noticeable gradient, diminishing from the muzzle to the rear end of the barrel.

## Conclusion

The innovative alginate-gelatine method has a dual usefulness, the topographic visualization of the staining inside firearm barrels and the differentiated trace securing and conserving. This allows for a further localised sampling in order to perform targeted molecular genetic analyses [15].
